# Hallux Valgus Management: An Update Based on National Institute for Health and Care Excellence (NICE) Guidelines

**DOI:** 10.7759/cureus.96642

**Published:** 2025-11-12

**Authors:** Burraq Imran, Stuart Jones, Rory G Middleton

**Affiliations:** 1 Medical School, University of Exeter, Exeter, GBR; 2 Orthopaedics and Trauma, Royal Cornwall Hospital, Truro, GBR; 3 Orthopaedics, Royal Cornwall Hospital, Truro, GBR

**Keywords:** ankle and foot, big toe, hallux valgus surgery, minimal invasive approach, orthopaedics

## Abstract

Hallux valgus, commonly known as a bunion, is a prevalent condition involving lateral deviation of the hallux (big toe). This may result in pain and functional impairment. Conservative methods such as orthoses, physiotherapy, and analgesia are first-line treatment. When conservative treatment fails, surgical intervention may be necessary, and traditionally, the gold standard of care has involved surgical intervention utilizing an open approach. However, recent National Institute for Health and Care Excellence (NICE) guidelines now recognizes the use of minimally invasive percutaneous surgical techniques as a viable option for Hallux correction alongside traditional open procedures. Open and minimally invasive techniques appear equivocal in their safety profiles with similar patient-reported outcome measures.

## Introduction and background

**Figure 1:**
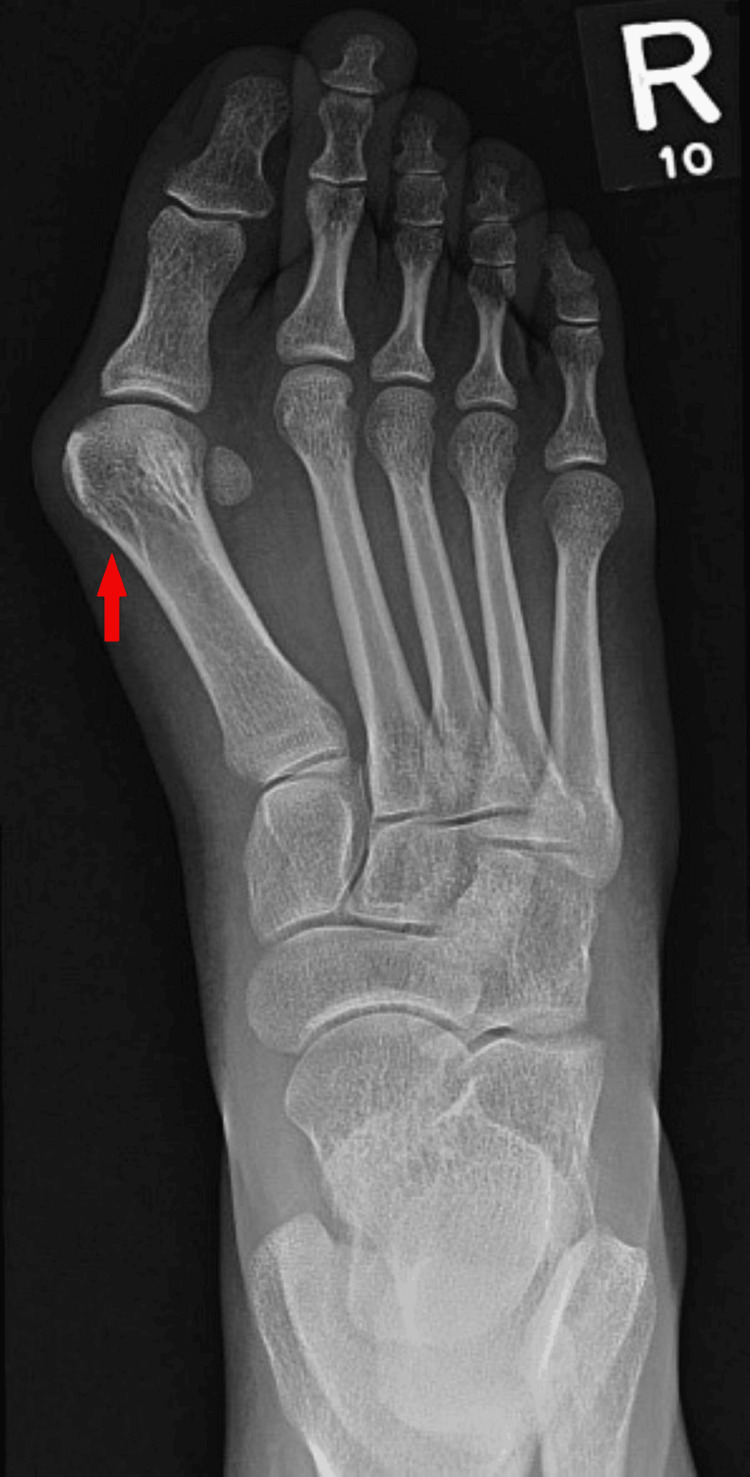
Right foot radiograph demonstrating severe hallux valgus deformity. Courtesy of Hani M Al Salam, Radiopaedia.org, rID: 12207. Adapted from Hani M Al Salam, Radiopaedia.org, available under the CC BY 4.0 International license (https://creativecommons.org/licenses/by/4.0/) [2].

Hallux valgus (HV), commonly known as a bunion, is a forefoot deformity of the big toe (hallux) which occurs when the proximal phalanx deviates laterally relative to a medial deviation of the first metatarsal head (Figure 1). HV is often a painless deformity requiring no further intervention [1]. Conservative treatments are aimed at symptom relief but do not prevent or slow down deformity progression.

While surgery can correct deformity, most guidelines would advocate that it is not indicated in asymptomatic patients and has its own referral criteria and risk profile [3].

Symptomatic patients typically present with a gradual onset of sharp pain at the metatarsal phalangeal (MTP) joint that is worse on weight bearing. As HV deformity progresses, the frequency, duration, and severity of pain tend to increase [1]. While conservative treatments such as exercises, orthotic devices, and pain management strategies are used during initial treatment, surgical intervention may be considered when these measures fail to provide adequate symptom relief [4-5].

Surgical correction of HV aims to alleviate pain and improve foot function through restoration of the proper alignment of the hallux [6]. Both open and minimally invasive approaches are described in the literature [7].

Historically, open surgery has been the preferred method of surgical intervention. There are various open surgical techniques available for HV treatment. For mild HV, a distal first metatarsal (MT) osteotomy (cutting and reorienting of bone) may be performed, most commonly through the chevron technique. In moderate to severe cases, proximal MT osteotomy is preferred and is often performed in combination with distal MT osteotomy [8].

Recent National Institute for Health and Care Excellence (NICE) guidelines have recognized the use of minimally invasive surgery (MIS) as an option for HV treatment [5].

MIS of HV was first described as early as the 1940s in the United States. Podiatrists were trying to work around restrictive laws surrounding which procedures they were able to perform [9]. A popular technique at the time was the Kramer osteotomy, later modified by Peter Bösch and OT New to become known as the subcapital osteotomy (SCOT) technique [10]. This procedure has since been the origin of all subsequent minimally invasive HV surgical techniques [11].

A first-generation percutaneous technique called the Reverdin-Isham procedure, which was first described in 1981, involves an intra-articular oblique and incomplete osteotomy of the head of the MT [12-13].

Currently, third-generation techniques are modifications of chevron-type osteotomies that incorporate the use of screws for added stability and have shown promising results [12].

This review aims to summarize the current evidence on open versus minimally invasive HV surgery based on NICE guidelines.

Inclusion criteria

This narrative review of the literature was conducted using the databases PubMed and Google Scholar, as well as institutional guidelines from the British Orthopaedic Association (BOA) and NICE. The search covered publications from 2009 to 2024, with additional landmark studies included where relevant. The search terms used were “hallux valgus,” “minimally invasive surgery,” and “open surgery,” combined using Boolean operators (e.g., AND, OR). Only full-text articles published in English and reporting on human adult populations were considered. Systematic reviews, meta-analyses, randomized controlled trials, retrospective cohort studies, and case series were included. Studies were excluded if they did not report relevant radiological or clinical outcomes.

To ensure transparency and assess study quality, included studies were appraised using established tools: AMSTAR for systematic reviews and meta-analyses and MINORS for non-randomized studies [14-15].

Epidemiology

HV is a common condition affecting around 23% of the adult population aged 18-65 years and 36% of those aged over 65. The prevalence is higher in women and those who wear shoes or heels when compared to the barefoot population [1,16-18].

Etiology

The etiology of HV is not well understood but is thought to be multifactorial in nature (Table 1). Structural factors that have shown the strongest correlation include a greater first Intermetatarsal angle, a longer MT, a round MT head, and the degree of lateral sesamoid displacement [19-20].

**Table 1:** Causes of hallux valgus. Created using Microsoft Word (version 2502 Build 16.0.18526.20168, Microsoft Corporation, Redmond, WA). Sources: [21-23].

Anatomical	Inflammatory	Other
Abnormal foot mechanics	Rheumatoid arthritis	Neurogenic
Abnormal MTP anatomy	Medial bursitis	Hereditary
Joint hypermobility	MTP synovitis	Idiopathic

History

In HV deformity, the proximal phalanx pronates and deviates laterally, while the MT head deviates medially, resulting in a hallux valgus angle (HVA) greater than 15°, with 0-15° considered normal (Figure 1). While many patients do not experience pain, those who do typically present with chronic medial forefoot pain at the first MTP joint, which worsens with movement. There may occasionally be pain in the other toes as well. This is usually due to either an altered gait because of the HV deformity or hammer toe deformity [6]. HV symptoms often become more frequent and intense in nature as the deformity progresses [1]. Other less common symptoms that affect patients include blisters, ulceration, interdigital keratosis, and skin irritation adjacent to the deformity [24].

Symptomatic HV can have a significant impact on patients' quality of life, making it important to treat promptly [1,25]. Surgery is not known to definitively prevent progression or recurrence, and a positive predictor for recurrence is age at the time of first surgery [26]. Therefore, conservative measures should be exhausted before progressing to surgery.

Examination

When examining HV deformity, it is important to not only evaluate the hallux itself but also the other toes and check the neurovascular integrity of the foot. A *look, feel, move* approach, along with a gait assessment, is a common way of approaching the examination for suspected HV (Table 2).

**Table 2:** Guide for examination of suspected hallux valgus. Created using Microsoft Word (version 2502 Build 16.0.18526.20168, Microsoft Corporation, Redmond, WA). Sources: [1,6]. MTP, metatarsal phalangeal

Step	Action
Look	Skin changes: Check for blisters, ulceration, and any skin irritation adjacent to the deformity. Foot position: Inspect the general alignment of the first metatarsal. Other deformities: Observe for hammertoe deformities, pes planus (flatfoot), or other visible abnormalities. Assess overall lower leg alignment.
Feel	Palpation: Press on the medial forefoot, particularly the first MTP joint, to assess for pain. Check for pain in other toes as well. Pulses: Assess dorsalis pedis and posterior tibial pulses. Sensation: Check for any sensory deficits in the foot to rule out nerve involvement.
Move	Range of motion: Assess the first MTP joint for crepitus, pain, or limited movement. Ligamentous integrity: Check for laxity or contractures in the first metatarsal. Deformity correction: Test passive correction of the hallux (especially internal rotation) to evaluate the reducibility of the deformity. Evaluate Achilles: Check for tightness in the Achilles tendon. Also, evaluate for any subtle changes in the patient's gait.

## Review

Investigations

While the initial assessment of HV involves conducting a thorough history and examination, the primary mode of investigation is a plain radiograph of the foot [19]. 

**Figure 2:**
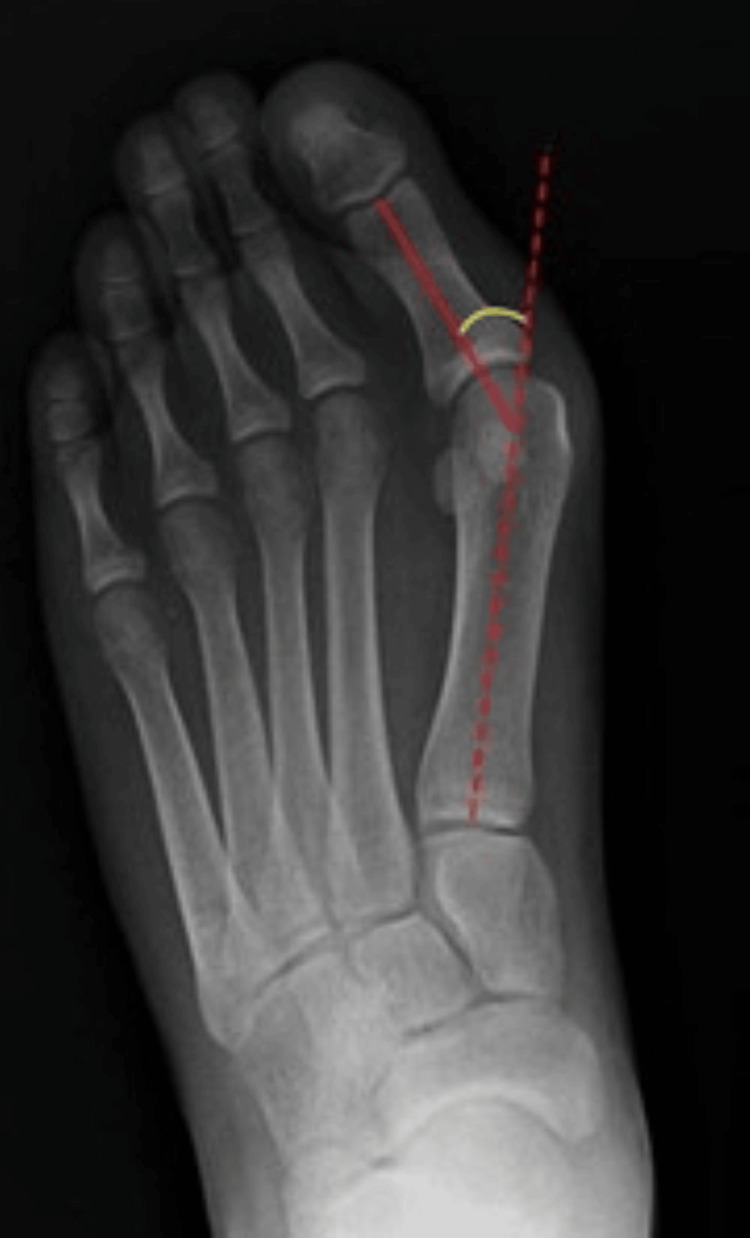
Anteroposterior (AP) foot radiograph demonstrating the measurement of the hallux valgus angle (HVA), with values less than 15° considered within the normal range. Courtesy of Samir Benoudina, Radiopaedia.org, rID: 42447. Adapted from Samir Benoudina, Radiopaedia.org, available under the CC BY 4.0 International license (https://creativecommons.org/licenses/by/4.0/) [31].

**Figure 3:**
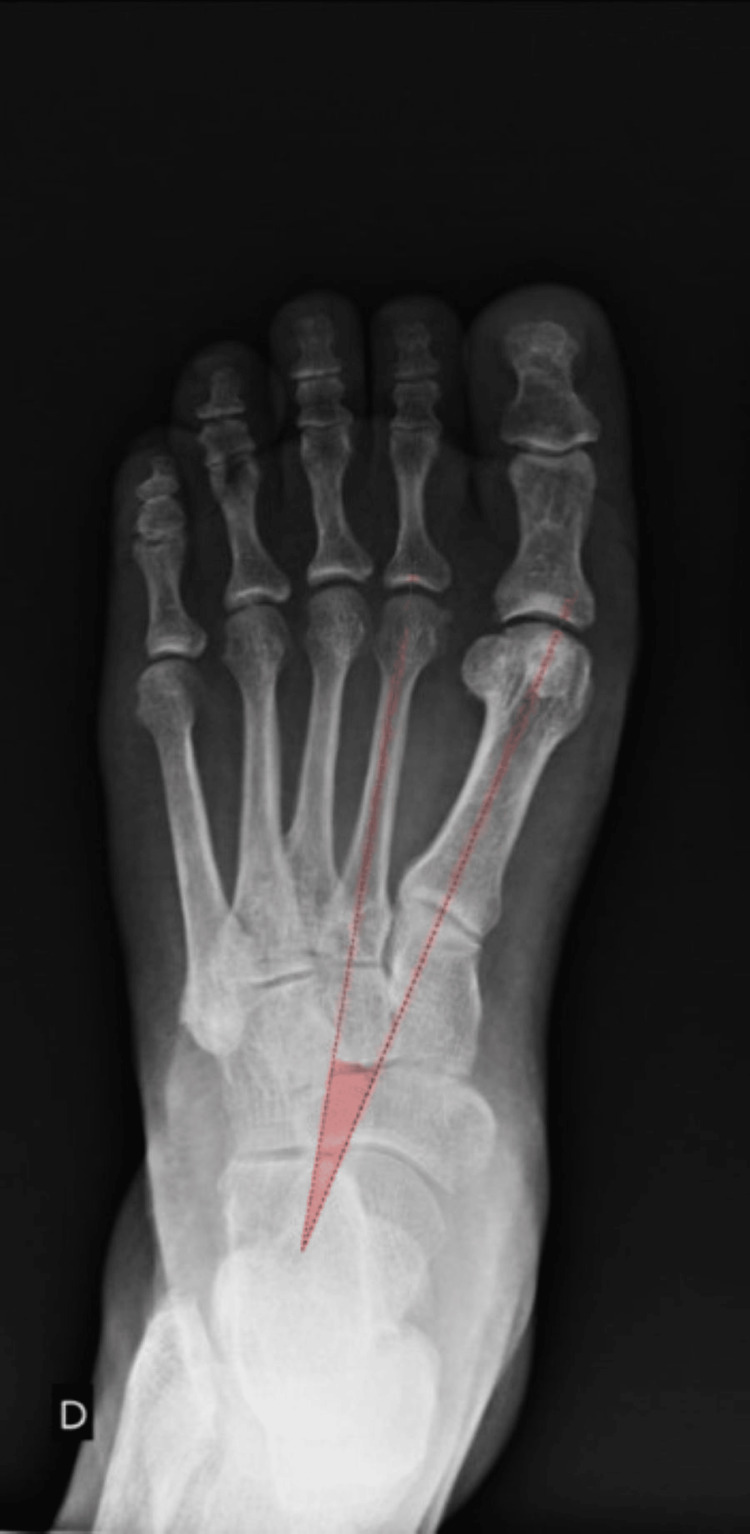
Anteroposterior (AP) foot radiograph demonstrating the measurement of the first–second intermetatarsal angle (IMA), with values less than 9° considered within the normal range. Courtesy of Leonardo Lustosa, Radiopaedia.org, rID: 91174. Adapted from Leonardo Lustosa, Radiopaedia.org, available under the CC BY 4.0 International license (https://creativecommons.org/licenses/by/4.0/) [32].

Weight-bearing anteroposterior (AP), lateral, and sesamoid views are essential for diagnosis. NICE uses the HVA and intermetatarsal angle (IMA) to sub-classify the severity of the deformity (Table 3). The AP view can be used to reliably measure the HVA and 1-2 IMA (Figures 1-3) [27-28]. The hallux valgus interphalangeal (HVI) angle is also important, as a high HVI angle can sometimes require multiple corrective procedures. Weight-bearing CT scans may also be used if further imaging is required [29]. Weight-bearing imaging provides a true representation of the deformity under physiological load, as non-weight-bearing views often underestimate the degree of angular displacement seen in functional stance.

**Table 3:** Severity scale for hallux valgus (HV) deformity. Patients must meet at least one of the criteria in the rows to be diagnosed. Created using Microsoft Word (version 2502 Build 16.0.18526.20168, Microsoft Corporation, Redmond, WA). Courtesy of the National Institute for Health and Care Excellence (NICE), available at https://www.nice.org.uk/guidance/ipg789. Explicit permission for use was obtained from NICE [30].

Severity	HVA angle (°)	IMA angle (°)
Normal	0-15	0-9
Mild	15-20	9-14
Moderate	20-40	14-20
Severe	40+	20+

Classification

Management

Conservative: Initial management of symptomatic HV involves conservative measures. It is vital to adopt a shared decision-making approach where patients and providers come up with treatment options together, considering the patients’ personal circumstances and treatment goals. Patients should be educated on their condition, as well as receive a footwear assessment and relevant analgesia. From here, patients may be provided with further options such as offloading orthotics. Another common treatment is physiotherapy to improve patient balance, proprioception, and core stability. However, it should be noted that current evidence supporting physiotherapy as a stand-alone treatment for HV is limited. Some patients may also be referred for therapeutic injections. However, this is usually only indicated if inflammation or arthritis is suspected or if the patient is unfit for surgery. This method of treatment is contraindicated if an infection is suspected [3].

Surgical: Surgery is usually considered only after conservative measures have been exhausted. The decision to operate is an important one; the BOA Commissioning Group recommends that the following criteria be met before proceeding with surgery, and emphasizes that surgery should never be performed for cosmetic reasons alone (Table 4) [3].

**Table 4:** Referral criteria for hallux valgus surgery. Courtesy of the British Orthopaedic Association (BOA), available at https://www.boa.ac.uk/static/8ccdae1a-c9ce-4938-8f7c61747f33f752/painful%20deformed%20great%20toe.pdf. Explicit permission for use was obtained from the BOA [3].

Referral criteria for surgery
Deteriorating symptoms
Failure of appropriate conservative measures after three months
Persistent pain and disability not responding to up to 12 weeks of non-surgical treatments
Patients must be prepared to undergo surgery, understanding that they will be out of sedentary work for two to six weeks and physical work for two to three months, and they will be unable to drive for six to eight weeks (two weeks if the left foot and driving an automatic car).
Age, gender, smoking, obesity, and comorbidity should not be barriers to referral.
Patients with significant comorbidities should have treatment that optimizes these conditions before referral.
Patients who are not suitable for surgery should be referred for a complex care package.

Open surgical techniques

An open approach to surgery has historically been preferred. Patients are placed supine with either an ankle block or general anesthesia, and a tourniquet is applied proximally to the medial malleolus. Three main techniques have been historically used.

**Figure 4:**
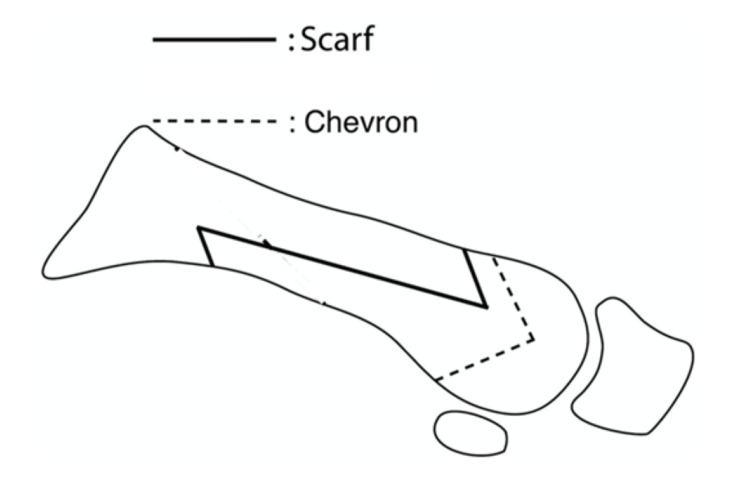
Position of cuts in a distal chevron osteotomy and scarf osteotomy Image from https://musculoskeletalkey.com/chapter-6-forefoot-pathology/, available under the CC BY 4.0 International license (https://creativecommons.org/licenses/by/4.0/) [36].

Distal first metatarsal chevron osteotomy is often used for mild HV. This technique involves the lateral displacement of the metatarsal head to reduce the IMA. A medial incision is made over the first metatarsophalangeal joint (MTPJ). The bursa is excised, and a V-shaped osteotomy is performed, allowing the metatarsal head to be shifted laterally toward the second toe to restore alignment and reduce the IMA (Figure 4). This can be performed at both the proximal and distal ends of the bone. In a recent meta-analysis, Clemente et al. reported that proximal chevron osteotomies achieved a mean IMA correction of 1.08° greater than distal chevron osteotomies, with typical correction ranges of around 9.5°-11° and 8.5°-9.5°, respectively [33]. The study did not report on recurrence rates of the respective surgeries.

Postoperatively, both active and passive physiotherapy exercises are able to commence the day after surgery [33,34]. 

A Scarf osteotomy, also known as a Z osteotomy, is another popular method used to treat moderate to severe HV. Although it can be utilized for mild HV, it is more invasive than the chevron osteotomy. A scarf osteotomy utilizes a similar approach on the medial side of the foot over the first MTPJ but involves making a Z-shaped cut on the first metatarsal, thus allowing lateralization of the metatarsal head to reduce the IMA, as well as rotational realignment with subsequent screw stabilization [35].

**Figure 5:**
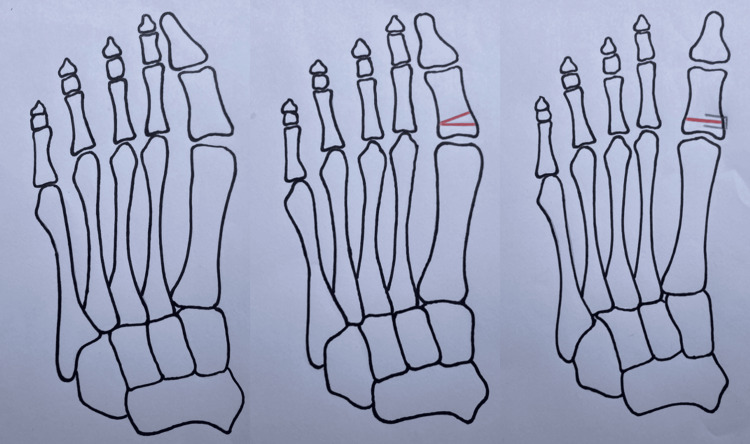
An Akin medial wedge osteotomy demonstrating removal of a bony wedge, resulting in straightening of the hallux. Figure illustration courtesy of Claire E. Cooper.

An Akin osteotomy used in moderate to severe HV is a closing medial wedge osteotomy of the proximal phalanx of the great toe (Figure 5). It is rarely used in isolation, more commonly utilized in conjunction with a scarf osteotomy procedure. It aims to help correct the metatarsophalangeal angle. Following the osteotomy and correction of alignment, it is then secured with a staple.

Malviya et al. found that both Scarf and Scarf-Akin procedures had similar functional and radiological outcomes. However, they also found that there was a higher risk of revision if scarf osteotomy was performed alone [36].

Minimally invasive techniques

Minimally invasive surgery is usually performed as a day case operation, under local or general anesthesia. The patient is placed in the supine position, and low-dose X-ray monitoring or endoscopic images are used throughout the procedure to guide incision sites and resection. In contrast to traditional open techniques that use a saw to perform the osteotomy, a burr is utilized, which allows for smaller incisions to be made. Following removal of the bunion, a metatarsal osteotomy is performed and secured with a plate and/or screws. After this, the incisions are closed, and a dressing is applied to facilitate healing [33]. Surgeons should be aware of the associated learning curve for minimally invasive techniques, as well as the small but notable risk of radiation exposure from intraoperative imaging.

In June 2024, the NICE Interventional Procedure Guidance committee reviewed current literature about minimally invasive surgery. A rapid review of literature involving two systematic reviews, three randomized controlled trials, three retrospective cohort studies, and three case series was performed. When comparing MIS with open procedures, several outcome measures can be used, including the Manchester-Oxford Foot Questionnaire (MOXFQ) score for quality of life; the American Orthopedic Foot and Ankle Society Hallux Metatarsophalangeal-Interphalangeal (AOFAS HMI) score for pain, joint function, and alignment; and the Visual Analog Scale (VAS) pain score for pain, in addition to complication rate, recurrence rate, and number of reoperations. They concluded that minimally invasive and open procedures have a similar efficacy and risk profile, with comparable patient-reported outcomes and recovery times for all severities of deformity [33].

Postoperative care

To ensure maximum benefit from the surgery, some restrictions are placed on patient activities postoperatively. Patients are advised to rest and keep their feet elevated for at least two weeks, avoid driving for six to eight weeks, stay off work for two to 12 weeks, and refrain from sports for three to six months. Patients should also be advised that following surgery, their toes might feel weaker or stiffer, may not be perfectly aligned, and they may still feel some residual pain in their feet [37]. Patients should also be scheduled for a minimum of three outpatient appointments with experienced foot and ankle physicians, with repeat radiographs being taken to assess healing during these appointments. Patients should also be advised that recurrence of deformity occurs in 8%-15% of patients (Table 5) [38].

**Table 5:** Postoperative complications of hallux valgus surgery. Created using Microsoft Word (version 2502 Build 16.0.18526.20168, Microsoft Corporation, Redmond, WA). Sources: [38].

Postoperative complications
Infection
Numbness and tingling
Nonunion formation
Chronic regional pain syndrome
Recurrence
Overcorrection (Hallux varus)
Screw irritation
Blood clots (pulmonary embolism or deep venous thrombosis)

Key studies 

The recent NICE MIS Hallux Guidance Update, upon which this paper was inspired, was based on the analyses of 1,975 patients from two systematic reviews and meta-analyses, three RCTs, three retrospective cohort studies (one with propensity score matching), and three case series. Of these 1,975 patients, 680 patients had minimally invasive percutaneous surgical techniques with internal fixation, and 303 patients had open osteotomy procedures. The primary efficacy outcomes, VAS and AOFAS scores, alongside the safety profile, were key considerations. These are outlined briefly in Table 6.

**Table 6:** Summary of key studies and outcome measures. Sources: [34,39-43]. Created using Microsoft Word (version 2502 Build 16.0.18526.20168, Microsoft Corporation, Redmond, WA). RCT, randomized controlled trial; HV, hallux valgus; MIS, minimally invasive surgery; OC, open chevron; HVA, hallux valgus angle; IMA, intermetatarsal angle; MTPJ, metatarsophalangeal joint; MICA, minimally invasive chevron-Akin; SA, scarf-Akin; POO, percutaneous oblique osteotomy; AOFAS, American Orthopedic Foot and Ankle Society; VAS, visual analog scale; MOXFQ, Manchester–Oxford Foot Questionnaire

First author, date	Study design	Inclusion criteria	Patients and sex (female:male)	Intervention	Follow-up	Efficacy outcomes	Safety outcomes
Dragosloveanu et al., 2022 [39]	RCT	Patients older than 20 years old, when conservative treatment had failed, with moderate valgus deformity (HVA between 20° and 40° and an IMA between 11° and 16°)	*n* = 50 patients with moderate HV	Percutaneous MIS chevron osteotomy (MIS group) compared with OC osteotomy (OC group)	1 year	Preoperative Open: VAS: 7.1 AOFAS: 61.4 MIS: VAS: 7.6 AOFAS: 65.7; six-month follow-up Open: VAS: 0.8 AOFAS: 79.4 MIS:VAS: 0.2 AOFAS: 85.6	Increased average radiological exposure time in the MIS group.
			Percutaneous MIS chevron osteotomy group: *n* = 24				Screw removal at 3 months due to tissue irritation: MIS osteotomy group: 12.5% Open group: 3.8%
			Open Chevron osteotomy group: *n* = 26				Metatarsalgia at six months in one open group patient
Kaufmann et al., 2019, 2020 [40-41]	RCT	Adults with HV who were scheduled to have a distal chevron osteotomy between January 2012 and August 2013. In all, nonoperative treatment had failed before surgery	*n* = 47 patients with HV	Percutaneous or MIS chevron osteotomy compared with OC osteotomy		Preoperative; Open: VAS: 6 AOFAS: 66.5 MIS: VAS: 5 AOFAS: 65; 12 weeks follow-up Open: VAS: 1 AOFAS: 83.5 MIS: VAS: 1 AOFAS: 85	Screw removal due to soft tissue irritation: Open: 4.5% MIS: 48%
			MIS chevron osteotomy: *n* = 25 feet				
			OC: *n* = 22 feet				
Kaufmann et al., 2020 [42]	Retrospective propensity score matched cohort study	Patients who had primary, unilateral MICA osteotomy for symptomatic HV between 2016 and 2018 for persistent painful bunion with or without metatarsalgia after	*n* = 60 patients with symptomatic HV	MICA osteotomy screw compared with open SA osteotomy (control). Other concomitant procedures were also done in a few cases	2 years	Preoperative Open: VAS: 5.6 AOFAS: 53.2; MIS: VAS: 5.5 AOFAS: 54.3; 6-month follow-up; Open: VAS: 1.2 AOFAS: 79.3; MIS: VAS: 1.2 AOFAS: 79.8	
			MICA osteotomy group: *n* = 30				
			Open SA osteotomy group: *n* = 30				
Guo et al., 2021 [43]	Retrospective cohort study	Patients above 18 years with painful HV and failed conservative treatment, and HV correction had operative treatment via Percutaneous oblique osteotomy (POO) or OC osteotomy techniques.	*n* = 112 feet (99 patients with HV)	Intervention: POO Control: OC osteotomy Akin osteotomy done in 50 (15 in POO and 35 in OC group) Weil osteotomy in 39 (16 in POO and 23 in OC group)	2 years	Preoperative Open: VAS: 7.29, AOFAS: 43.7 MOXFQ - Walking and standing score: 49.8 Social interaction score: 58.6 Pain score: 49.8; MIS: VAS: 7.63, AOFAS: 46.4 MOXFQ - Walking and standing score: 50.3 Social interaction score: 56.9 Pain score: 51.6	8.3% complication rate in the percutaneous group versus 12.5% in the open group
			POO group: *n* = 48 feet				
						2-year follow-up Open: VAS: 2.56, AOFAS: 79.5 MOXFQ - Walking and standing score: 15.2 Social interaction score: 17.6 Pain score: 22.7; MIS: VAS: 1.55, AOFAS: 85.2 MOXFQ - Walking and standing score: 13.5 Social interaction score: 13.3 Pain score: 15.8	
			OC osteotomy group, *n* = 64 feet				
Clemente et al., 2022 [34]	Meta-analysis	*n* = 985 across 10 studies. Average age 32.7-54 years. Stratification by gender was reported in eight studies, with a percentage of males ranging from 0 to 13.7%	*n* = 496 distal chevron osteotomy	Open distal chevron osteotomy, proximal chevron osteotomy, scarf osteotomy, Lingdren osteotomy, Percutaneous distal metatarsal osteotomy, Wilson, Mitchell.	12- 56.4 months.	Open distal chevron osteotomy was associated with a mean IMA correction of 2.18° greater than the scarf procedure.	
			*n* = 102 proximal chevron osteotomy				
			*n* = 164 scarf osteotomy			Proximal chevron was associated with a mean IMA correction of 1.08° greater than the distal chevron. The AOFAS assessment showed a 3.2-point greater improvement in favor of the Lingdren group compared with the distal chevron osteotomy.	
			*n* = 82 Lingdren osteotomy				
			Percutaneous distal metatarsal osteotomy, *n* = 29			No statistically significant difference between open and percutaneous distal osteotomy was reported at 12-week follow-up.	
			Wilson *n* = 42, Mitchell *n* = 60.				

Key points

HV is a common condition affecting around 23% of adults aged 18-65. Patients with HV commonly present with chronic, worsening medial-sided pain in the forefoot, alongside functional impairment. Approximately 3% of men and 11% of women live with HV. Diagnosis involves weight-bearing plain radiographs of the foot from AP, lateral, and sesamoid views. Initial treatment for HV involves analgesia, orthotics, physiotherapy, and therapeutic injections. Surgery is the only permanent treatment for HV and can now be done either open or through minimally invasive techniques.

## Conclusions

HV is a common condition affecting a large percentage of the adult population. The condition can significantly impact a patient's quality of life due to pain and impaired function of the foot. For this reason, it is imperative to treat promptly. Weight-bearing plain radiographs are crucial for the diagnosis of HV. Initial management should include conservative measures such as orthotics use alongside physiotherapy, with joint injections indicated in some patients. When these measures are not enough, surgery may be indicated. Both open and minimally invasive surgery have been shown to have comparable results in terms of patient-reported outcomes, with equivalent safety profiles and recovery times. To minimize risks and complications, it is crucial that, whichever surgical approach is chosen, the surgeon is competent and trained in that procedure.
